# Accelerating the Biodegradation of High-Density Polyethylene (HDPE) Using *Bjerkandera adusta* TBB-03 and Lignocellulose Substrates

**DOI:** 10.3390/microorganisms7090304

**Published:** 2019-08-31

**Authors:** Bo Ram Kang, Soo Bin Kim, Hyun A Song, Tae Kwon Lee

**Affiliations:** Department of Environmental Engineering, Yonsei University, Wonju 26493, Korea

**Keywords:** high-density polyethylene (HDPE), *Bjerkandera adusta*, laccase, Raman spectroscopy, Scanning electron microscopy

## Abstract

High-density polyethylene (HDPE) is a widely used organic polymer and an emerging pollutant, because it is very stable and nonbiodegradable. Several fungal species that produce delignifying enzymes are known to be promising degraders of recalcitrant polymers, but research on the decomposition of plastics is scarce. In this study, white rot fungus, *Bjerkandera adusta* TBB-03, was isolated and characterized for its ability to degrade HDPE under lignocellulose substrate treatment. Ash (*Fraxinus rhynchophylla*) wood chips were found to stimulate laccase production (activity was > 210 U/L after 10 days of cultivation), and subsequently used for HDPE degradation assay. After 90 days, cracks formed on the surface of HDPE samples treated with TBB-03 and ash wood chips in both liquid and solid states. Raman analysis showed that the amorphous structure of HDPE was degraded by enzymes produced by TBB-03. Overall, TBB-03 is a promising resource for the biodegradation of HDPE, and this work sheds light on further applications for fungus-based plastic degradation systems.

## 1. Introduction

Plastics are artificially synthesized organic polymers that are inexpensive and durable. These materials are widely used and have become indispensable in modern society [1]. In Korea, the production of plastics increased sharply from 5.6 million tons per year in 2011 to around 6.9 million tons per year in 2015 [2]. The most commonly used plastics in Korea are polyethylene terephthalate, polypropylene, polystyrene, and polyethylene (PE). PE is the most consumed plastic in the world, and 140 million tons of it are produced each year [3]. PE is classified into high-density polyethylene (HDPE) and low-density polyethylene (LDPE), depending on the polymerization methods used in its synthesis [4].

HDPE generally remains in the environment due to its durability [5]. However, after being broken down into small particles by extrinsic factors, it can be bioaccumulated into living organisms or the environment. To reduce HDPE-induced biorisks, sustainable and cost-effective technologies are needed to treat HDPE. A number of studies have reported HDPE degradation using fungal enzymes (e.g., laccase, and peroxidases). Ascomycota fungal species (e.g., *Aspergillus*) have been reported to degrade HDPE in liquid medium under laboratory conditions [6]. The Basidiomycota are also well known for producing extracellular oxidative enzymes such as laccase and peroxidases, and some of these enzymes have been reported to be capable of degrading plastics [7]. Nevertheless, the Basidiomycota have rarely been studied in terms of plastic degradation. In addition, fungal enzyme production associated with lignocellulose substrates was scarcely studied for HDPE degradation.

In this study, we investigated the potential of *Bjerkandera adusta* to degrade HDPE. *B. adusta* demonstrates a highly effective ability to produce laccase when decomposing a lignocellulose substrate [8]. *B. adusta* TBB-03 was cultured under two different conditions with lignocellulose substrates, carbon-replaced liquid medium and solid-state fermentation (SSF), to accelerate HDPE degradation. The chemical and structural changes of HDPE after fungal treatment was analyzed using Raman spectroscopy and scanning electron microscopy (SEM). This study presents the use of Basidiomycota species for HDPE degradation as a novel concept in tackling the ever-increasing problem of the environmental accumulation of plastic.

## 2. Materials and Methods

### 2.1. Isolation and Identification of Fungi

Wild fungi were collected from the Ohgap Mountains, North Chungcheong Province, South Korea. Each mycelia sample was isolated using sterile tweezers and transferred onto Potato Dextrose Agar in order to obtain a pure culture. An agar plug (6 mm in diameter) was taken from the edge of the fungal growth area and transferred onto modified indicator agar containing 2,2′-azino-bis(3-ethylbenzthiazoline-6-sulfonic acid) (ABTS) [9]. When oxidized by extracellular oxidative enzymes, the ABTS became visible as a green-colored halo on the indicator agar. Genomic DNA of the fungal isolate, indicated by the widest diameter of green halo, was extracted using a FastDNA™ SPIN KIT (MP Biomedicals, USA) following the manufacturer’s instructions. PCR amplification of the internal transcribed spacer (ITS) region of specimens was performed using primers ITS-1F and ITS4, according to White et al. [10]. The purified amplicon was sequenced at Macrogen (Seoul, South Korea), and the resulting sequence was deposited in GenBank under accession number MK806486. A phylogeny was constructed by performing the neighbor-joining method with MEGA7 [11].

### 2.2. Preparation of HDPE

Commercial HDPE (0.05 mm thick) plastic bags were purchased from a domestic market (Clean bag, Clean Wrap, South Korea). Sheets were cut into small strips of 5 × 5 cm and washed with 70% ethanol then distilled water. Surface-treated samples were placed in 5 mL snap tubes and autoclaved at 120 °C for 10 min.

### 2.3. Enzyme Production and Measurement of Enzyme Activity

A mycelial inoculum was prepared as described by Blánquez et al. [12]. To increase enzyme activity, CuSO_4_, ferulic acid, vanillic acid, veratryl alcohol, and 2, 5-xylidine were tested as inducers. The inducers were dissolved in methanol, sterilized by filtration, and added to minimal medium (glucose 10 g, KH_2_PO_4_ 1 g, MgSO_4_∙7H_2_O 0.05 g, CaCl_2_∙2H_2_O 0.013 g, and yeast extract 0.025 g in L^−1^) to give a final concentration of 0.5–2 mM, except CuSO_4_, which had a final concentration of 10–100 μM. Also, 1 mL of each supernatant was harvested at given time intervals.

Five lignocellulose substrates with different lignin proportions were purchased from a domestic online market. The lignin contents of ash (*Fraxinus rhynchophylla*), fir (*Abies holophylla*), mesquite (*Prosopis spicigera*), hickory (*Carya tomentosa*), and oak (*Quercus alba*) were obtained from our previous research [13] using the acetyl bromide method as described by Moreira-Vilar et al. [14], and the values were 11.8 (± 2.9), 12.6 (± 2.0), 15.9 (± 1.1), 4.3 (± 2.0) and 15.2 (± 0.5) (mg lignin/g cell-wall), respectively. Lignocellulose substrates were cut in size 0.7–1.0 cm, washed with distilled water, and dried at 60 °C for 24 h. Two grams of each substrate were injected as the sole carbon source into 250 mL Erlenmeyer flasks containing 100 mL of 1% peptone solution (pH 4.5) before being inoculated with 1% (*v*/*v*) mycelium inoculum. The fungal cultures were incubated at 25 °C and 120 rpm for 10 days. The supernatants were filtered using 0.2 μm PES syringe filters (Whatman, Germany) and used directly in measurement of enzyme activity. Laccase activities were determined by oxidation of 0.5 mM ABTS at 420 nm in 0.1 M acetate buffer at pH 4.5 (ε_420_ = 36,000 M^−1^ cm^−1^) [15]. Manganese peroxidase (MnP) activities were calculated according to the oxidation of 7 mM MnSO_4_ at 238 nm in 0.1 M tartrate buffer (pH 5) containing 0.05 mM H_2_O_2_ (ε_310_ = 6500 M^−1^ cm^−1^) [16]. Lignin peroxidase (LiP) activities were determined from the rate of oxidation of 2 mM veratryl alcohol at 310 nm in 0.1 M tartrate buffer (pH 3) containing 0.4 mM H_2_O_2_ (ε_310_ = 9300 M^−1^ cm^−1^) [17]. One unit of activity (U) was defined as the amount of enzyme required to oxidize 1 μmol of product per min.

### 2.4. Degradation Assays

Three different culture conditions were used for the degradation assays: (i) basal medium; (ii) carbon-replaced liquid medium with lignocellulose substrates; and (iii) SSF. Malt extract medium (ME; malt extract 20 g, peptone 1 g L^−1^, pH 4.5) was selected as the basal medium for this study. Sterilized ash wood chips (20 g L^−1^) were injected in ME instead of malt extract for the carbon-replaced liquid medium (LM). The ME and LM were prepared in 250 mL Erlenmeyer flasks containing 100 mL of broth and inoculated with 1% (*v*/*v*) mycelium inoculum. For SSF, 100 g of sterilized ash wood chips were placed in 250 mL Erlenmeyer flasks and inoculated with 10% (*v*/*v*) mycelium inoculum. ME and LM were incubated with HDPE samples at 25 °C and 120 rpm for 90 days, whereas there was no agitation for SSF. HDPE samples without additional treatments served as controls.

### 2.5. Raman Spectroscopy

Measurement of Raman spectra were performed with a Confocal Raman Imaging System (XperRam35V, Nanobase, South Korea), equipped with 3 port excitation 532 nm DPSS laser (LTL-532RL, Leading tech, South Korea), microscope body (Olympus BX43, Olympus, Japan), spectrometer (XPE-35 VPHG, Nanobase, South Korea) and charge-coupled device (Atik 428EX, Atik, Portugal). The polarization rotator rotated 0–180 degree continuously using a zero order half-wave plate and the polarizations were collected with extinction ratio> 200:1 and transmission> 83%. The laser power at the sample was 2 mW. Spectrometer grating was 1800 gr/mm. The total acquisition time for each spectrum was 500 ms. Raman spectra were collected from 10 locations across the surface of the HDPE sample.

### 2.6. Scanning Electron Microscopy Observation

The changes in surface morphology of the control and treated HDPE samples were investigated using SEM (Quanta 250 FEG, FEI Co, Salt Lake, UT, USA). The HDPE samples were coated with a thin layer of Pt for 30 s and affixed to the sample holder. The acceleration voltage was 20 kV, and the current was 15 mA.

### 2.7. Statistical Tests

All statistical analyses were performed in R [18]. One-way analysis of variance (ANOVA) followed by Duncan’s test was used to determine statistically significant differences between the groups at *p* < 0.05. Substrate specificity was assessed by calculating Spearman’s correlation coefficient.

## 3. Results and Discussion

### 3.1. Isolation and Identification of Laccase-Producing Fungal Strains

**Figure 1:**
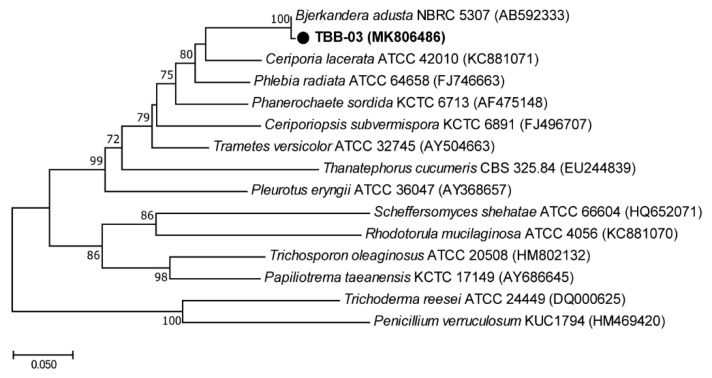
Phylogenetic tree for TBB-03 and related strains based on internal transcribed spacer (ITS) gene sequences. Phylogeny of TBB-03 generated from neighbor-joining analysis of ITS sequences. The scale bar corresponds to 0.05 substitutions per nucleotide position.

Each fungal isolate was screened for their ability to produce oxidative enzymes for 3 days at 25 °C. Fungal isolate TBB-03 showed the largest green halo zone (diameter > 65 mm), which indicated the presence of extracellular oxidative enzymes and was, therefore, selected for the HDPE degradation studies. Molecular identification of the TBB-03 was performed by ITS sequence analysis. The ITS sequence showed a 99% similarity to the sequence of *Bjerkandera adusta* (Figure 1). *B. adusta* is a Basidiomycota fungus and plays an ecologically important role in the global carbon cycle by decomposing wood and leaf litter [19]. Oxidative enzymes, including manganese peroxidase (MnP), lignin peroxidase (LiP), and laccase, from *B. adusta* are highly effective at decomposing lignocellulose substrates, recalcitrant pollutants, and dyes [20,21]. In addition, *B. adusta* has been reported to decompose compact discs consisting of aromatic polymers [7].

### 3.2. Enzyme Production During the Cultivation on Media Containing Different Lignocellulose Substrates

TBB-03 was able to produce extracellular laccase, whereas no LiP and MnP activity was detected on ME medium in this study. Shaking has been reported to improve laccase production, but MnP and LiP production is higher in stationary cultures than under agitation [22]. To increase the yield of laccase, known inducers, including CuSO_4_, ferulic acid, vanillic acid, veratryl alcohol, and 2, 5-xylidine, were injected into the cultures [23]. Unexpectedly, most of the inducers did not significantly stimulate laccase production (0.6–27.0 U/L) after 10 days. Vanillic acid (1 mM) induced laccase production (44.3 U/L), but this value was relatively modest compared with laccase activity in ME alone (69.1 U/L). These results indicate that the addition of inducers did not improve the enzyme production level and activity. Simple inducers such as metal ions, aromatic compounds and nutrients are known to regulate laccase expression in a synergistic or antagonistic way [24]. TBB-03 seemed to require a more complex combination inducer than a simple inducer.

**Figure 2:**
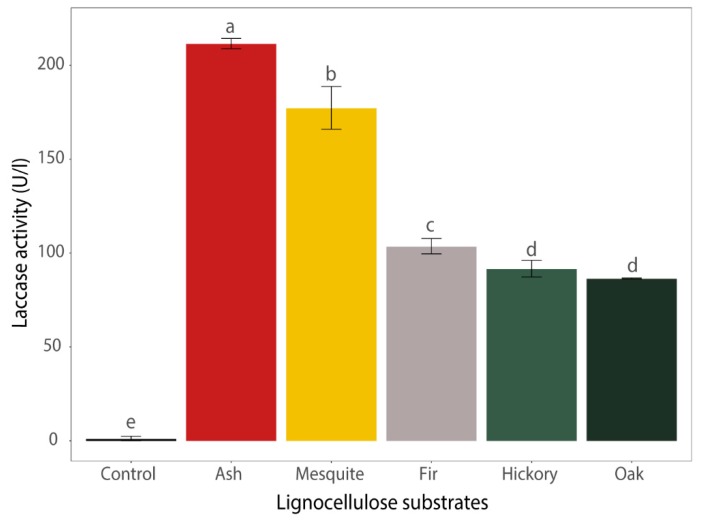
Laccase activity in culture supernatant incubated with various lignocellulose substrates. Ash, mesquite, fir, hickory, and oak were tested at 20 g L^−1^ as sole carbon sources for laccase production. Significant differences at *p* < 0.05 between the laccase activity were calculated using Duncan’s multiple range test.

In order to evaluate the effect of introducing complex lignocellulose substrates on laccase production, five different types of lignocellulose substrates were injected onto the fungal cultures. The greater yields of enzyme obtained after the injection of lignocellulose substrates are consistent with previous reports involving white rot Basidiomycota fungi, including *Trametes versicolor* and *Pleurotus ostreatus* [8,25]. A comparison of laccase production rates using ash, mesquite, fir, hickory, and oak wood chips as the sole carbon source was conducted. All lignocellulose substrates significantly increased laccase activity (86.3–211.4 U/L after 10 days) (Figure 2). The highest level (211.4 U/L) of laccase activity was observed under ash wood chips treatment. The lignin contents of lignocellulose substrates had no direct effects on laccase activity. The results were consistent with the previous results showing that *Trametes versicolor*, model Basidiomycota species, showed highest laccase activity with ash as the sole carbon source [15]. Hence, ash wood chips were selected as the sole carbon source for further studies. It has been reported that laccase has a wide substrate specificity, but enzyme production is regulated by secondary metabolites generated in the substrate [26]. Rice straw has been found to be the most suitable substrate for growth and enzyme production of *B. adusta* SM46 when compared with other sources of lignocellulose substrates, including wood meal, kapok fiber, and pulp waste [27]. Bagewadi et al. tested several agricultural residues to enhance production of laccase and optimized SSF condition with wheat bran [28]. These results support the idea from previous literatures of using lignocellulose substrates not only as growth sources but also as natural enzyme inducers with lignin-derived compounds [29,30,31].

### 3.3. Chemical, Structural, and Morphological Changes of HDPE

The experiment on HDPE degradation by TBB-03 was carried out using two methods of ash wood chips addition (LM and SSF). SSF has previously been an attractive and cost-effective approach with sufficient oxygen supply and no additional substrate injection requirements. However, Basidiomycota species have been reported to show different enzyme activities depending on the use of SSF or LM in long-term experiments [32,33]. Generally, the total activity of laccase and the number of isoforms decreased when cultures were grown in SSF, but the MnP and LiP may be produced additionally since SSF do not have an agitation processes [22]. After treatment with both the LM and SSF conditions, the structural changes of HDPE were analyzed by Raman spectroscopy and SEM.

Structural analysis is the most important parameter in identifying structural changes during degradation. Lignocellulolytic enzymes, such as laccase in PE degradation, are usually involved in reactions with free radicals, resulting in chain cleavage, cross-linking, and formation of carbonyl groups [34]. Raman spectroscopy is sensitive to local molecular environments and, as a consequence, has been widely applied to investigate the interactions between macromolecules during PE degradation [35,36]. Raman analysis of degraded HDPE provides a close view of C–C anti-symmetric stretching at 1064 cm^−1^, C–C symmetric stretching at 1130 cm^−1^, CH_2_ twisting (crystalline) at 1295 cm^−1^, CH_2_ bending (crystalline) at 1416 cm^−1^, CH_2_ bending (amorphous trans) at 1440 cm^−1^, CH_2_ bending (amorphous) at 1460 cm^−1^, and C–H stretching at 2825–2970 cm^−1^. Details of the assignments for the Raman bands are listed in Table 1 [37].

**Figure 3:**
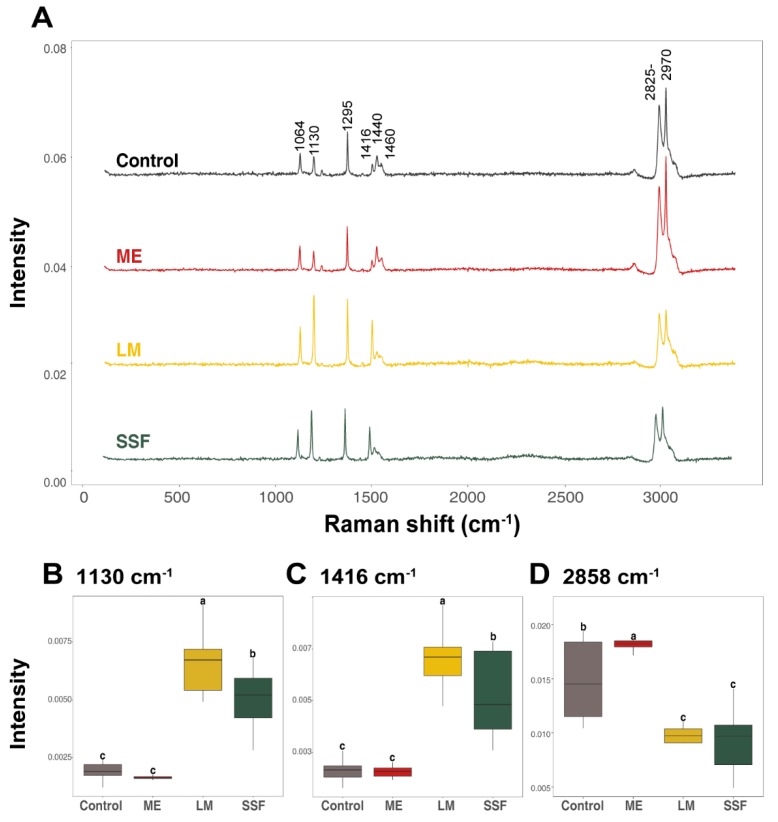
Discrimination of the control and treated HDPE after 90 days of treatment with the fungal isolate TBB-03. (**A**) represents the Raman spectra of each condition. Raman spectra were collected from 10 different locations, and an averaged spectrum was used for the analysis. Relative intensity plots of peaks at (**B**) 1130 cm^−1^, (**C**) 1416 cm^−1^, and (**D**) 2858 cm^−1^. Significant differences at *p* < 0.05 between peaks were calculated using Duncan’s multiple range test.

The structural changes were observed in the HDPE degraded by TBB-03 using Raman microspectroscopy, as depicted in Figure 3A. The heights of the Raman bands were remarkably different when TBB-03 was treated with ash wood chips compared to the control. The effects of the HDPE degradation from two different treatment methods were negligible. Unlike TBB-03 treatment with lignocellulose substrates, HDPE in ME were almost unchanged (slightly increased at 2825–2970 cm^−1^). These results suggest that lignocellulose substrate addition, which could provide a variety of carbon sources as well as inducers, were required to degrade HDPE.

There was no formation or disappearance of Raman bands in HDPE. The peaks at 1130 cm^−1^, 1295 cm^−1^, and 1416 cm^−1^, corresponding to C–C symmetric stretching, CH_2_ twisting, and CH_2_ bending from crystalline, respectively, showed an increase, whereas the peaks at 2825–2970 cm^−1^ decreased (Figure 3B–D). These findings are consistent with previous studies. Previous studies have shown that these trends are due to an increase in crystallinity and the contraction of interchain distances, but a decrease in amorphous chains causes the degradation [36]. The sharp increase in crystallinity after fungal treatment with ash wood chips could be explained by the chemicrystallization process, which occurs in the initial periods of plastic degradation through chain scission in the amorphous phase and a decrease in the interlamellar amorphous layer until inactivation of plasticity causes brittleness [38]. Similar spectral changes have been observed during biodegradation or environmental stress cracking [39,40]. These results indicate that TBB-03 cultured in the presence of ash wood chips could change the chemical structure of HDPE. In addition, there is no significant differences between the treatment methods because the biodegradation takes place by limiting to an amorphous layer of the HDPE.

**Figure 4:**
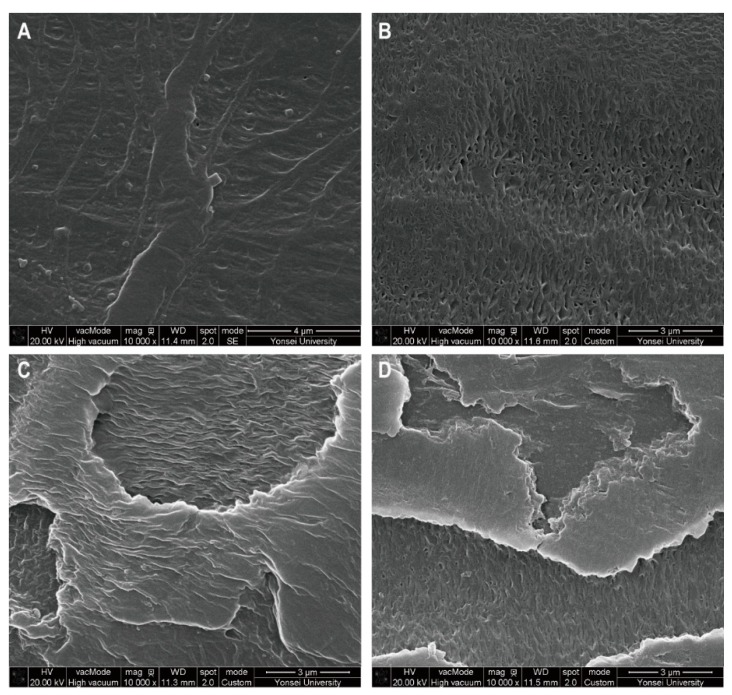
SEM micrographs of control and treated HDPE samples at 10,000× magnification. (**A**) Control, (**B**) malt extract medium (ME), (**C**) liquid medium (LM), and (**D**) solid-state fermentation (SSF).

SEM analysis was used to confirm that the surface of HDPE became physically weak after fungal treatment. There are negligible morphological changes in control (no treatment) or fungal treatments with ME, respectively (Figure 4A,B). After 90 days of co-incubation with TBB-03 and ash wood chips, the formation of pits and cracks, which are typical events associated with the biodegradation process, were observed on the surface of the HDPE using SEM (Figure 4C,D). At low magnification, the degradation on the surface of the HDPE was seen to occur in most areas (Figure S1). Previous studies have reported similar morphological changes on HDPE degraded by Ascomycota fungal species, including *Aspergillus* and *Penicillium* [6,41]. Basidiomycota species have been reported to produce similar results with LDPE or modified PE [42]. The SEM results showed that the cracks appeared to be minimal on the ME (Figure 4B) but became conspicuous when ash wood chips were added. These results could explain why the Raman bands at 2825–2970 cm^−1^ increased slightly compared with the raw HDPE temporarily when the amorphous layer in the PE started to decrease [38]. The increase in the rate of HDPE degradation when the ash wood chips was added as the substrate may be due not only to the increase in the amount of laccase but also to the increase in laccase activity due to a mediator resulting from lignin decomposition. A wide range of mediators from lignocellulose substrates are oxidized by laccase and can act as strong oxidants by producing radical intermediates [43,44]. The presence of mediators also allows laccase to oxidize chemical structures that would otherwise remain untouched due to selectivity [45]. These results showed that the morphology change of HDPE was significant in the presence of TBB-03 and ash wood chips.

## 4. Conclusions

This study demonstrates that HDPE is degraded by exposure to *B. adusta* TBB-03 in the presence of a lignocellulose substrate. To the best of our knowledge, this is the first research to propose HDPE degradation by *B. adusta* with ash wood chips and show the ensuing chemical, structural, and morphological changes using Raman spectroscopy and SEM. The quantitative analyses (e.g., biodegradation rates) on the various types of the plastics including HDPE needs further validation using experimentation with standardized or field conditions for the waste management or designing the treatment plants. Despite these limitations in this study, the presented results suggested that *B. adusta* TBB-03 is a promising sources for the plastic biodegradation and waste management processes.

## Figures and Tables

**Table 1:** Vibrational phase and modes of the Raman spectrum of high-density polyethylene (HDPE).

Raman shift, cm^−1^	Phase	Mode
1064	Crystalline, trans chain	ν_as_ (C–C)
1130	Crystalline, trans chain	ν_s_ (C–C)
1295	Crystalline	τ (CH_2_)
1416	Crystalline (orthorhombic)	δ (CH_2_)
1440	Amorphous trans (intermediate)	δ (CH_2_)
1460	Amorphous	δ (CH_2_)

ν: stretching; ν_s_: symmetric stretching; ν_as_: anti-symmetric stretching; τ: twisting; δ: bending.

## Supplementary Materials

The following are available online at https://www.mdpi.com/2076-2607/7/9/304/s1. Figure S1: SEM micrographs of control and treated HDPE samples at 1000× magnification. (A) Control, (B) ME, (C) LM, and (D) SSF.
