# Supplementary material for: Accelerating the Biodegradation of High-Density Polyethylene (HDPE) Using Bjerkandera adusta TBB-03 and Lignocellulose Substrates
Source: Microorganisms. 2019 Aug 31;7(9):304. doi: 10.3390/microorganisms7090304 (PMC6780323; doi:10.3390/microorganisms7090304)
Supplement: Supplementary file 1 [file microorganisms-07-00304-s001.pdf]

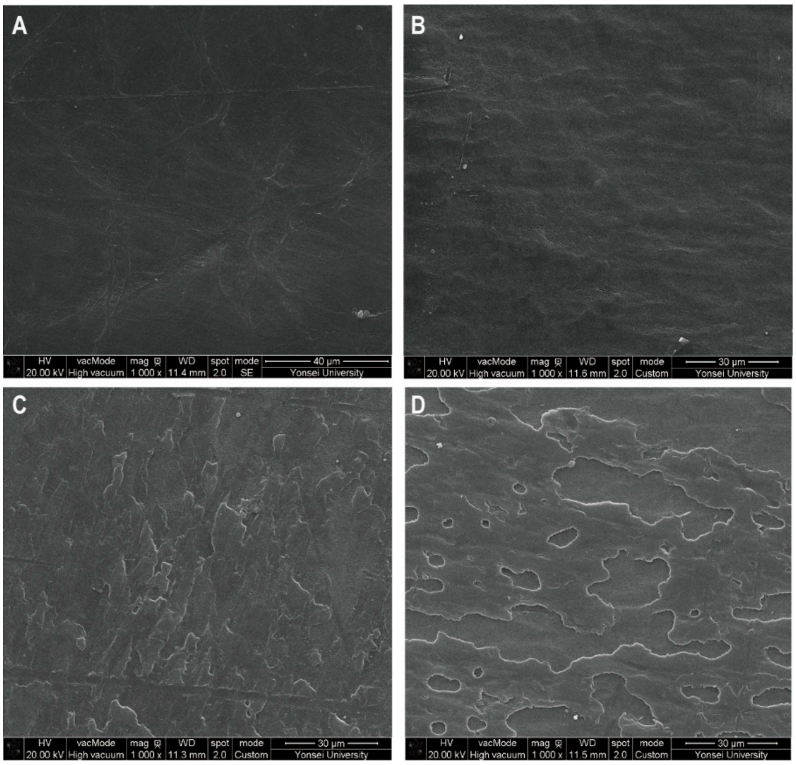

Figure S1. SEM micrographs of control and treated HDPE samples at 1,000× magnification. (A) Control, (B) ME, (C) LM, and (D) SSF.
